# Social networks of men who have sex with men and their implications for HIV/STI interventions: results from a cross-sectional study using respondent-driven sampling in a large and a small city in Tanzania

**DOI:** 10.1136/bmjopen-2016-012072

**Published:** 2016-11-18

**Authors:** Michael W Ross, Markus Larsson, Jerry Jacobson, Joyce Nyoni, Anette Agardh

**Affiliations:** 1 Program in Human Sexuality, Department of Family Medicine, University of Minnesota, Minneapolis, Minnesota, USA; 2 Division of Social Medicine and Global Health, Department of Clinical Sciences, Lund University, Malmö, Sweden; 3 Independent Consultant, Los Angeles, California, USA; 4 Department of Sociology and Anthropology, University of Dar es Salaam, Dar es Salaam, Tanzania

**Keywords:** homosexual, Africa, Tanzania, Social networks, STIs

## Abstract

**Objective:**

Men who have sex with men (MSM) in sub-Saharan Africa remain hidden and hard to reach for involvement in HIV and sexually transmitted infection (STI) services. The aim of the current study was to describe MSM social networks in a large and a small Tanzanian city in order to explore their utility for peer-based healthcare interventions.

**Methods:**

Data were collected through respondent-driven sampling (RDS) in Dar es Salaam (n=197) and in Tanga (n=99) in 2012 and 2013, using 5 and 4 seeds, respectively. All results were adjusted for RDS sampling design.

**Results:**

Mean personal network size based on the number of MSM who were reported by the participants, as known to them was 12.0±15.5 in Dar es Salaam and 7.6±8.1 in Tanga. Mean actual RDS network size was 39.4±31.4 in Dar es Salaam and 25.3±9.7 in Tanga. A majority (97%) reported that the person from whom they received the recruitment coupon was a sexual partner, close friend or acquaintance. Homophile in recruitment patterns (selective affiliation) was present for age, gay openness, and HIV status in Dar es Salaam, and for sexual identification in Tanga.

**Conclusions:**

The personal network sizes and existence of contacts between recruiter and referral indicate that it is possible to use peer-driven interventions to reach MSM for HIV/STI interventions in larger and smaller sub-Saharan African cities. The study was reviewed and approved by the University of Texas Health Science Center's Institutional Review Board (HSC-SPH-10-0033) and the Tanzanian National Institute for Medical Research (NIMR/HQ/R.8a/Vol. IX/1088).

**Strengths and limitations of this study**

- This is the first study of social networks and network characteristics of men who have sex with men (MSM) in Africa.
- This study describes the organisation of social networks and implications for disease transmission, community-level screening and treatment.
- The data show large and well-connected networks that are relatively easily accessed in both a large and a smaller city.
- Respondent-driven sampling however misses those not associated with a network and ‘loner’ MSM.
- The sample is disproportionately composed of younger MSM.

## Introduction

Men who have sex with men (MSM) in sub-Saharan Africa remain hidden and hard to reach for involvement in HIV and sexually transmitted infection (STI) services. This is particularly troubling, given the high HIV and STI rates that have been reported across the continent.^1^ ^2^ Criminalising laws, stigma and discrimination in the healthcare system, and poverty are some of the root causes as to why this population continues to be marginalised from the healthcare system. Therefore, accessing MSM outside the healthcare system remains critical in order to reach this population.

Applying a network-level analysis to MSM social networks could contribute to an increased understanding of some of the underlying factors influencing HIV dynamics in this population.^3^ Studies suggest that the social support gained from being a member of a network plays a key role for gaining access to HIV-related information, including knowledge of HIV-testing sites^4^ that networks provide role-modelling of safer sexual behaviors^4–6^ and that they offer emotional support for both HIV-positive as well as HIV-negative MSM.^7^ ^8^ Conversely, observations have been made regarding the negative influence of networks with low levels of normative support for safe sex on individual members' involvement in high-risk sexual behaviours.^9^ ^10^ In the context of sub-Saharan Africa, studies have shown that MSM social networks constitute critical vehicles for transmission of HIV-related information. Kajubi *et al*^11^ found that 67% of the respondents in Kampala, Uganda, primarily received information about HIV from their friends. In a study from Botswana, 42% of the interviewed MSM said that friends provided them with HIV/AIDS information.^12^

Social networks can also be used to recruit and reach MSM for research and intervention purposes, and one approach that has emerged during the previous decade is respondent-driven sampling (RDS).^13^ ^14^ RDS is initiated with a convenience-sampling approach by selecting seed individuals who are asked to distribute a predetermined number of coupons across their respective network to members who in turn recruit from their network, so called waves.^13^ The underlying assumption is that a sample becomes independent from its initial bias if the chain referral, or number of waves, is large enough.^14^ Estimation methods that weight for potential under-representation and over-representation are used to present unbiased population estimates.^15^ While a social networks analysis generally relies on egocentric data focusing on the ties, attributes and density of personal networks of all available actors in a population, Wejnert^16^ demonstrated in a study on racial integration in the US that RDS collected data were sufficient to make inferences regarding social networks.

While there have been previous RDS studies of MSM in East Africa, our current knowledge is still relatively limited regarding the details of the social networks involved in this region. An increased understanding of the structure and composition of MSM networks such as the sizes, types and forms, is crucial for peer-based interventions that leverage these networks to engage MSM in HIV/STI prevention and treatment. This might be particularly relevant in settings where geographically defined gay venues are absent due to laws and societal stigma, and possibilities to reach a larger population are limited. The current study examines characteristics of networks of MSM, using data from a previously conducted survey, where RDS recruiting was used to estimate prevalence of HIV and STIs in this population in two Tanzanian cities.^2^ The aim is to describe MSM social networks in a large and a small Tanzanian city in order to explore their utility for peer-based healthcare interventions. It was hypothesised that (RDS) networks would be significantly larger in size in the larger city with more specialised networks compared with the smaller city due to their different general population size, hence requiring a proportionally smaller number of seeds to achieve minimum sample size in comparison with the smaller city.

## Methods

### Study design, sampling and recruitment

The present study used network data from a larger cross-sectional parent study^2^ previously conducted in Dar es Salaam in 2012, which has a population of ∼4.3 million, and in Tanga in 2013, a provincial city north of Dar es Salaam with a population of about 273 000.^17^ The required sample size in each city was calculated prior to starting the parent study and was based on achieving a sufficient power to discriminate between MSM with and without HIV.^2^ In Dar es Salaam the sample size was estimated at 200 MSMs, and in Tanga to 100 MSMs. The survey used RDS to recruit participants for the study. This sampling methodology has been found useful in settings where the target group remains hard to reach.^18^ Since homosexual behaviours are illegal in Tanzania we anticipated that it would be difficult to reach MSM openly. This was addressed by recruiting participants through their own networks; each recruited respondent recruited further study participants from his personal network. Eligible participants were those above 18 years and who had sex with another man during the past 6 months.

Prior to the study start, discussions were held with a Community Advisory Board with representatives from the MSM group for orientation purposes, that is, what kind of information should be provided, training of the seeds, recruitment strategies. In each city we selected five seeds, which were of different ages (three seeds in each city below 30 years of age) and from various areas of the cities. They were informed about the study purpose and eligibility requirements, and were also given basic training as to how they should approach other study participants. Each seed was provided with three coupons, each with a unique identification number that linked the coupon to the recruiter. The seeds recruited three MSM each from their respective network, provided them with a coupon and accompanied them to the research site, where participants who met the eligibility criteria were given three new coupons to recruit from his network (so-called waves). In total six men in Dar es Salaam and five in Tanga were excluded due to ineligibility or due to inability to present a coupon.

To determine when the sample had reached convergence (when the variables stop changing^19^), we examined the RDS convergence plots for age and education. These variables had been selected as we anticipated that they would be associated with HIV and STI risk behaviour and network structure. The number of coupons was reduced as we approached the estimated sample size in each city, and the last wave of respondents was not provided with any coupons. Convergence for our two variables of interest (ie, age and education) had been reached at the time of the final wave.

### Procedure

Eligible participants were asked to complete a self-administered interview consisting of a structured questionnaire and some open-ended questions regarding their sexual history, network, STI symptoms and health-seeking behaviours. The interview was administered on a laptop assisted by a university-educated research assistant, trained in research interviewing, and required ∼30–40 min for completion. Prior to participation, oral informed consent with detailed information regarding the study aim, interview purpose, and the benefits/risks of participation was obtained in Swahili or English (Tanzania's two official languages), as preferred by participants. Participants were informed that their identity and confidentiality would be fully protected. During the interview, support for participants who had difficulty in understanding item(s) was provided by the research assistants, who read or explained the item(s). The same procedure was applied for those participants, who were unable to read, by reading the questions aloud. The interviews took place in a private house on a bus route, rented by the project team. Participants were given the option to conduct the interview either in English or in Swahili. Both versions of the questionnaire had been pre-assessed by a panel of English and Swahili speaking experts for accuracy, comprehension, and content-validity. Prior to the study, the Swahili version had been pilot-tested on five MSM for comprehension, clarity, and response range, and modified as appropriate. Each participant received 5000 Tanzanian shillings (TZS) (about 2.75USD) for study participation (primary incentive) and an additional 5000 TZS for each successful referral (secondary incentive). For the biological sampling component, which was voluntary, blood, urine and anal swab tests were taken to measure HIV type 1 (HIV-1), syphilis, hepatitis B, chlamydia, and gonorrhoea. Of the total sample of 300 MSMs, 38 declined testing (28 in Dar es Salaam and 10 in Tanga). (See ref. 2 for a thorough description of the biological sampling component).

### Measures

*Sexual identification* was assessed by the question ‘Do you consider yourself to be’ and the options were recorded as ‘gay/homosexual’, ‘straight/heterosexual’, ‘bisexual’, ‘undecided’, ‘other (specify)’, and ‘no response’. Two categories were created ‘gay’ and ‘bisexual’, respectively. Those, who reported ‘heterosexual’, ‘undecided’, ‘other (specify)’, and ‘no response’ were excluded from the analysis.

*Gay openness* was assessed on the basis of responses to the question ‘Does/do [name of social contact] know you have sex with men?’. Responses were obtained separately for 12 types of social contacts: ‘male friends who also have sex with other men’, ‘male friends who have sex with only women’, ‘female friends’, ‘wife’, ‘mother’, ‘father’, ‘brothers’, ‘sisters’, ‘coworkers’, ‘employers’, ‘doctors/nurses’, and ‘anyone else’. Gay openness was defined as 50% or more of the 12 items had a ‘yes’ response, and allocated a score of 1. Those who reported ‘yes’ to five or less items were given a score of 0. The cut-off point of gay openness was an arbitrary derived measure for the purpose of enabling binary homophile analyses.

*Personal network size* was based on the response to two questions: (1) ‘How many gay or bisexual men do you know personally, who are living in this city, are 15 years of age or older, you know their name and they know you?’ and (2) ‘How many of these [repeat number] gay or bisexual men have you seen in the past one month?’.

*Actual network size* was the mean number of connected respondents in each network divided by the number of networks, per city.

*Network connectivity* was determined by asking three questions: (1) ‘How would you best describe your relationship to your recruiter, ie, the person who gave you this coupon?’. The response alternatives were ‘a stranger, someone you met for the first time’, ‘someone you know, but not closely’, ‘a close friend, someone you know very well’, ‘a sexual partner’, ‘a family member or relative’, and ‘no response’. (2) ‘How often do you see your recruiter?’. The response alternatives were ‘every day’, ‘once a week’, ‘once a month’, ‘less than once a month’, and ‘no response’. (3) ‘About how long have you known your recruiter?’. The response alternatives were ‘<6 months’, ‘6 months to 1 year’, ‘1–2 years’, ‘>2 years’, and ‘no response’. ‘No response’ was excluded from the analysis.

*Homophile index*: In RDS theory homophile reflects the tendency of participants to recruit others with similar characteristics beyond random mixing. In the present study homophile was assessed using Heckathorn's^19^ index of affiliation and calculated using RDS analyst (RDS-II estimator) (RDS Analyst: Software for the Analysis of Respondent-Driven Sampling Data, Version 0.42 [program]. Los Angeles the Hard-to-Reach Population Methods Research Group (HPMRG), 2014) for measures of age, education, HIV status, STI status, sexual identification, sex work, experiences of stigma by the general community and by health professionals, and a derived index of openness regarding sexual orientation (gay openness). These measures were defined as follows.

*Age* was based on an open-ended response to the question ‘How old are you (completed years)?’ and examined both as ‘age group 1’ (18–21, 22–30 and 31–59 years) and as ‘age group 2’ (18–30 and 31–59 years) to enable more detailed analyses.

*Education*, was based on the question ‘What is the highest level of education you have attained?’ with the response alternatives were ‘never been to school’, ‘some primary school’, ‘completed primary school’, ‘some secondary school’, ‘completed secondary school’, ‘A level’, ‘O level’, ‘some tertiary school’, ‘completed tertiary school’ and ‘other (specify)’. Two groups of categories were created to enable more detailed analyses. ‘Education group 1’ contained ‘less than primary’ (‘never been to school’ and ‘some primary school’), ‘primary–secondary’ (‘completed primary school’, ‘some secondary school’ and ‘completed secondary school’) and ‘any tertiary or above’ (‘A level’, ‘O level’, ‘some tertiary school’ and ‘completed tertiary school’). ‘Education group 2’ contained ‘less than secondary’ (‘never been to school’, ‘some primary school’, ‘completed primary school’ and ‘some secondary school’) and ‘secondary or above’ (‘completed secondary school’, ‘A level’, ‘O level’, ‘some tertiary school’ and ‘completed tertiary school’). ‘Other (specify)’ was excluded from the analysis.

*HIV status* was based on the test of the biological sampling component for HIV-1 and coded as ‘HIV positive’ or ‘HIV negative’.

*STI status* was based on the test of the biological sampling component for syphilis, hepatitis B, *chlamydia*, and gonorrhoea and coded as ‘STI positive’ (for any or several positive results) or ‘STI negative’.

*Sex work* was based on the following question ‘Have you ever been paid money by someone, male or female, in exchange for oral or vaginal sex?’ and ‘Have you ever been paid money by someone, male or female, in exchange for anal sex?’ and the response alternatives were ‘yes’ and ‘no’. Participants who indicated ‘yes’ to any or both of these questions were categorised as ‘yes’ (ie, ever been paid in exchange for sex) and the rest as ‘no’ (ie, not been paid in exchange for sex).

*Stigma estimation* was based on the following questions: ‘How much stigma is there toward gay/homosexual men among the general community here?’ and ‘How much stigma is there toward gay/homosexual men among doctors and nurses?’, and measured on a five-point Likert scale with the following response alternatives ‘no stigma’, ‘minimal stigma’ ‘not much stigma’, ‘moderate stigma’ and ‘a lot of stigma’. These variables were then dichotomised by combining the first four alternatives as ‘no’ and the last alternative as ‘yes’.

### Data preparation

We reviewed the consistency of the data by comparing the study data set against the participant register (‘log’), and by examining RDS and coupon codes to verify the consistency (ie, that the data set and the logs contained identical data) of linkages between recruiters and referrals. One participant from Dar es Salaam with two referrals was missing from the data. For purposes of analysis, we assigned the ‘grandparent’ (that participant's recruiter) as the recruiter of these referrals. Three participants from Dar es Salaam were excluded from the analysis because their RDS codes were duplicated, and their correct codes could not be determined. One record in the Tanga data set was excluded because the participant could not be linked to a recruiter or any referrals and did not appear on the participant register. Following these changes, there were 197 Dar es Salaam and 99 Tanga participants remaining for analysis. n may vary because of missing values on some variables.

### Statistical analysis

A measure of personal network size is required for weighting of RDS data and is assumed to be proportional to the probability of selection into the study.^19^ We imputed network size at the city mean for 11 (6%) participants from Dar es Salaam, who had a missing response to either of these questions. For a further 11 (6%) Dar es Salaam and 1 Tanga participants, the reported network size was inconsistently low and was replaced with the number of their referral plus one representing their recruiter. For other measures missing values were excluded from the analysis of each respective measure and thus n may not sum to the total sample n.

We estimated population proportions using RDS Analyst (RDS-II estimator) (RDS Analyst. 2014) with 2000 bootstraps, which weights by inversing network size. The χ^2^ tests were calculated to assess the relationship between characteristics of participants and their recruiters. Significance level was set at 5% (two-tailed) for all analyses. Comparisons between continuous variables were made by Student's t-test for independent samples, and between ordinal variables, by χ^2^ test, with Yates correction for discontinuity where appropriate. Data preparation was performed in Stata V.12.0 (StataCorp. College Station, Texas, USA). Some bivariate sample comparisons were carried out using SPSS V.22.0 (Armonk, New York, USA: IBM Corp).

## Results

### Sociodemographic characteristics

Over half of the sample populations in both Dar es Salaam (56.3%) and Tanga (55.6%) were between 22 and 30 years old. Most respondents reported either primary or secondary school education. More respondents in Dar es Salaam, 65% identified themselves as gay or homosexual compared with Tanga (51.2%) (tables 1 and 2). Approximately 30% reported ever selling sex in Dar es Salaam, and 35% in Tanga. The mean percentage for gay openness was 54.3% in Dar es Salaam and 60.6% in Tanga. RDS-adjusted demographic characteristics for respondents in Dar es Salaam and Tanga are presented in tables 1 and 2, respectively.

**Table 1:** Characteristics of MSM in Dar es Salaam by crude and RDS-adjusted estimates

Variable	Category	n (%)	RDS-adjusted total % (95% CI)
Age group 1	18–21	62 (31.5)	31.2 (23.6 to 38.7)
	22–30	111 (56.3)	57.9 (46.5 to 69.3)
	31–59	24 (12.2)	10.9 (0.0 to 22.2)
Age group 2	18–30	173 (87.8)	89.1 (82.1 to 96.1)
	31–59	24 (12.2)	10.9 (3.9 to 17.9)
Education group 1	Less than primary	10 (5.1)	4.7 (0.0 to 10.1)
	Primary–secondary	176 (89.3)	90.3 (85.9 to 94.8)
	Any tertiary or above	11 (5.6)	5.0 (1.6 to 8.3)
Education group 2	Less than secondary	121 (61.4)	59.8 (50.7 to 68.9)
	Secondary or above	76 (38.6)	40.2 (31.1 to 49.3)
Sexual identification	Bisexual	66 (35.0)	44.6 (35.8 to 53.4)
	Gay/homosexual	124 (65.0)	55.4 (46.6 to 64.2)
Missing		7	
Ever been paid in exchange for sex	No	137 (69.5)	77.2 (67.4 to 87.0)
	Yes	60 (30.5)	22.8 (13.0 to 32.6)
Stigma estimation in general community	No	15 (7.6)	8.6 (3.7 to 13.5)
	Yes	182 (92.4)	91.4 (86.5 to 96.3)
Stigma estimation among doctors, nurses, etc	No	32 (16.3)	16.2 (8.6 to 23.8)
	Yes	164 (83.7)	83.8 (76.2 to 91.4)
Missing		1	
Openness about being MSM	Openness ≤0.5	90 (45.7)	33.1 (24.1 to 42.0)
	Openness >0.5	107 (54.3)	66.9 (58.0 to 75.9)
HIV infection (laboratory test)	HIV negative	118 (69.8)	82.0 (70.0 to 93.9)
	HIV positive	51 (30.2)	18.0 (6.1 to 30.0)
Missing		28	
Any STI infection excluding HIV (laboratory test)	STI negative	161 (82.0)	85.4 (76.5 to 94.3)
	STI positive	36 (18.0)	14.6 (5.7 to 23.5)

Continuous variables	Minimum	Maximum	Mean	Median	SD	n
Age	18	59	25	23	4.7	197
Openness about being MSM	0	1	0.48	0.5	0.28	197

MSM, men who have sex with men; RDS, respondent-driven sampling; STI, sexually transmitted infection.

**Table 2:** Characteristics of MSM in Tanga by crude and RDS-adjusted estimates

Variable	Category	n (%)	RDS-adjusted total % (95% CI)
Age group 1	18–21	31 (31.3)	35.9 (22.5 to 49.2)
	22–30	55 (55.6)	49.4 (37.0 to 61.9)
	31–59	13 (13.1)	14.7 (6.8 to 22.6)
Age group 2	18–30	86 (86.9)	85.3 (77.4 to 93.2)
	31–59	13 (13.1)	14.7 (6.8 to 22.6)
Education group 1	Less than secondary	64 (64.5)	66.4 (54.9 to 77.8)
	Secondary or above	35 (35.5)	33.6 (22.2 to 45.1)
Education group 2	Less than primary	5 (5.1)	3.5 (−3.6 to 10.6)
	Primary–secondary	91 (91.9)	92.8 (83.1 to 102.5)
	Any tertiary or above	3 (3.0)	3.7 (−2.3 to 9.7)
Sexual identification	Bisexual	46 (48.8)	54.4 (40.0 to 68.9)
	Gay/homosexual	48 (51.2)	45.6 (31.1 to 60.0)
Missing		5	
Ever been paid in exchange for sex	No	64 (64.6)	68.3 (58.6 to 78.1)
	Yes	35 (35.4)	31.7 (21.9 to 41.4)
Stigma estimation in general community	No	23 (23.2)	22.0 (10.2 to 33.9)
	Yes	76 (76.8)	78.0 (66.1 to 89.8)
Stigma estimation among doctors, nurses, etc	No	32 (32.3)	28.7 (17.7 to 39.7)
	Yes	67 (67.7)	71.3 (60.3 to 82.3)
Openness about being MSM	Openness ≤0.5	39 (39.4)	37.3 (23.9 to 50.6)
	Openness >0.5	60 (60.6)	62.7 (49.4 to 76.1)
HIV infection (laboratory test)	HIV negative	80 (88.9)	89.1 (81.4 to 96.8)
	HIV positive	10 (11.1)	10.9 (3.2 to 18.6)
Missing		9	
Any STI infection excluding HIV (laboratory test)	STI negative	32 (94.1)	99.0 (97.4 to 100.5)
	STI positive	2 (5.9)	1.0 (−0.5 to 2.6)
Missing		65	

Continuous variables	Minimum	Maximum	Mean	Median	SD	n
Age	18	45	25	23	5.16	99
Openness about being MSM	0	1	0.5	0.4	0.26	99

MSM, men who have sex with men; RDS, respondent-driven sampling; STI, sexually transmitted infection.

### Recruitment and network size

Five seeds in Dar es Salaam and four seeds in Tanga were productive in recruitment (table 3). The largest of the resulting recruitment chains accounted for 38–39% of referrals, such that no one chain dominated the sample in either study site. The largest chains reached up to six waves in Dar es Salaam and seven waves in Tanga (table 3). Based on number of persons in the participants' own networks, the mean reported personal network size was 12.0 (SD 15.5) in Dar es Salaam, and 7.6 (SD 8.1) in Tanga (df=265, t=3.2, p=0.000, where df is the number of persons in the network). The median reported personal network size was 6.0 in both cities (table 4). The mean actual RDS network size based on the number of networks in each city was 39.4 (SD 31.4) in Dar es Salaam and 25.3 (SD 9.7) in Tanga (df=7, t=0.86, p=0.42, where df is the number of separate networks). The actual RDS network median size was 37 (of five networks) in Dar es Salaam and 24.5 (of four networks) in Tanga. In both Dar es Salaam and Tanga, 25% of MSM reported that they knew fewer than four other MSM and 50% fewer than six other MSM (data not shown in table).

**Table 3:** Respondent-driven sampling (RDS) recruitment chains for men who have sex with men (MSM) in Tanzania

	Dar es Salaam		Tanga	
Recruitment chain (seed ID)	Number of referrals (%)	Number of waves	Number of referrals (%)	Number of waves
1	75 (39%)	5	36 (38%)	5
2	65 (34%)	5	28 (29%)	7
3	36 (19%)	6	19 (20%)	4
4	12 (6%)	2	12 (13%)	4
5	4 (2%)	2	–	–

**Table 4:** Recruiter and network characteristics

Variable	Dar es Salaam n (%)*	Tanga n (%)*
Mean and median reported personal network size	12.0, 6.0	7.6, 6.0
Mean and median actual RDS network size	39.4, 37	25.3, 24.5
Relationship to recruiter		
A stranger, someone you met for the first time	2 (1.5)	8 (8.0)
Someone you know, but not closely	66 (33.7)	28 (28.3)
A close friend, someone you know very well	124 (62.8)	61 (61.6)
A sexual partner	5 (2.5)	2 (2.0)
A family member or relative	–	–
How often do you see your recruiter?		
Every day	87 (44.2)	46 (46.5)
Once a week	92(46.7)	37 (37.7)
Once a month	13 (6.6)	14 (14.1)
Less than once a month	5 (2.5)	2 (2.0)
How long have you known your recruiter?		
<6 months	29 (14.7)	3 (3.0)
6 months to 1 year	21 (10.7)	15 (15.2)
1–2 years	31 (15.7)	38 (38.4)
>2 years	116 (58.9)	43 (43.4)

*Some ns may not sum to sample size due to missing data.

RDS, respondent-driven sampling.

### Network connectivity

An assumption of RDS is that referrals know their recruiters. Most participants reported that the person from whom they received the recruitment coupon was a sexual partner, a close friend or an acquaintance (195 (98%) in Dar es Salaam, 91 (92%) in Tanga), that they typically saw their recruiter at least once a month (192 (97%) in Dar es Salaam and 97 (98%) in Tanga), and that they had known their recruiter for 6 months or longer (168 (85%) in Dar es Salaam and 96 (97%) in Tanga), see table 4. The number of participants who reported that they would have recruited the same person who had recruited them for the study was 192 (97%) in Dar es Salaam and 98 (99%) in Tanga (data not shown in table).

### Homophile

**Figure 1:**
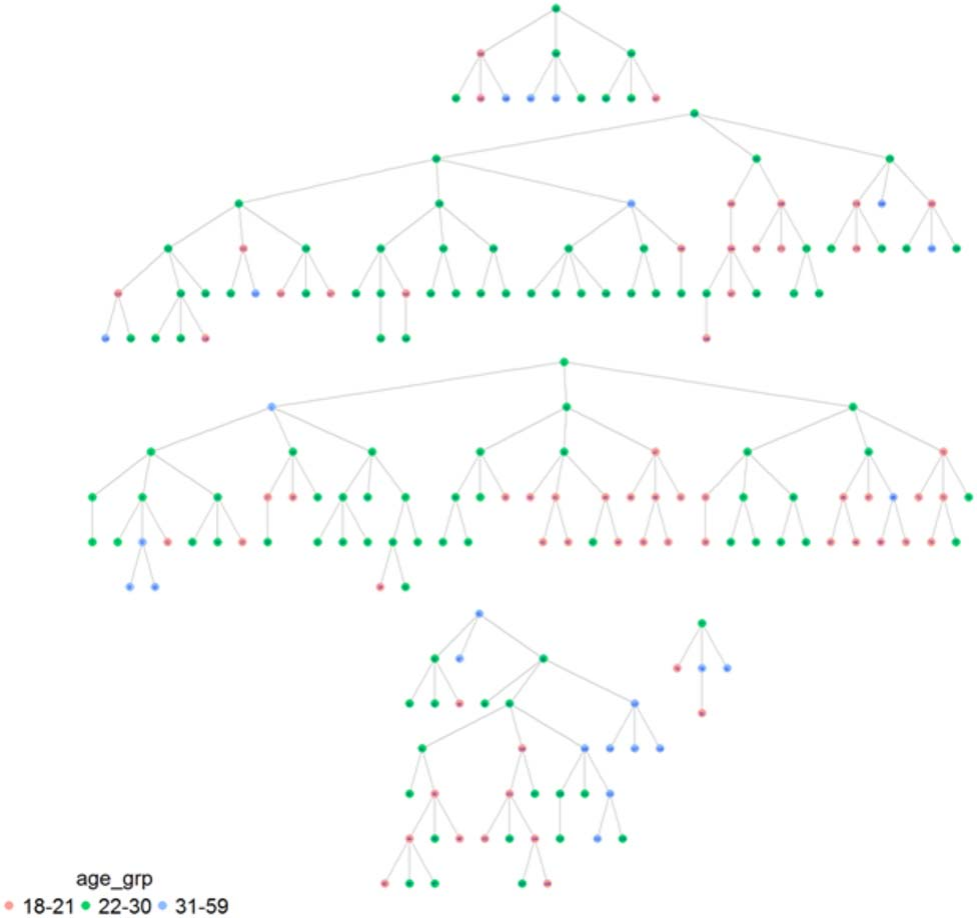
MSM network in Dar es Salaam by age.

**Figure 2:**
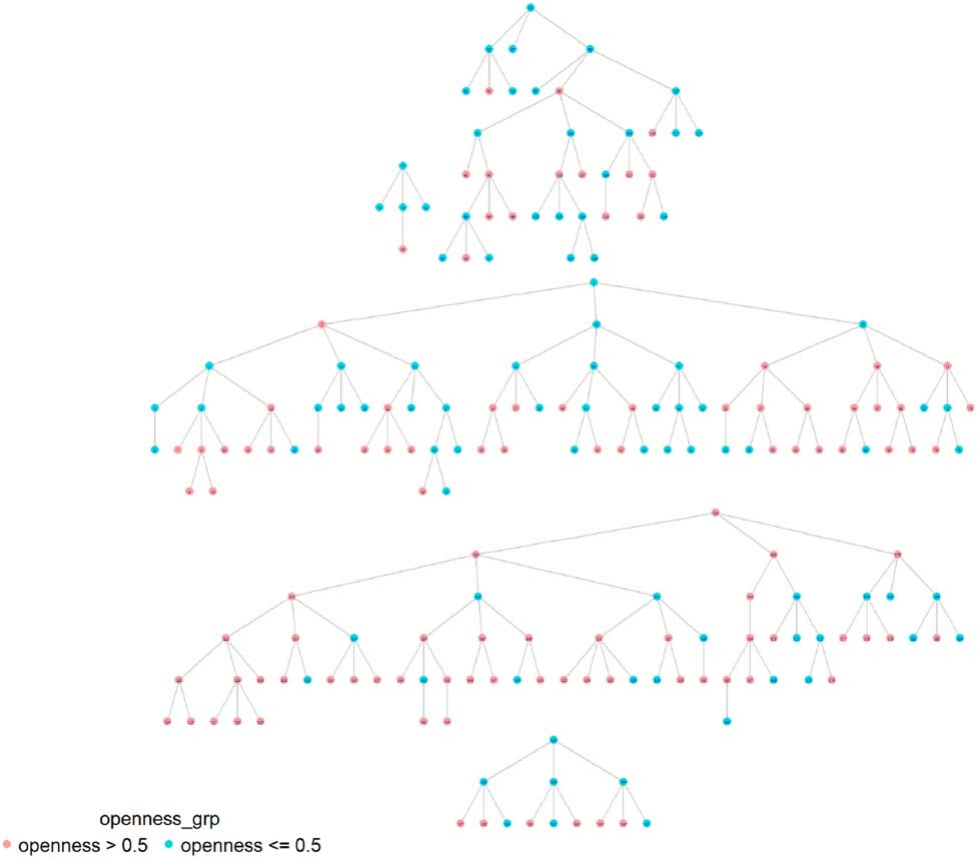
MSM network in Dar es Salaam by gay openness.

**Figure 3:**
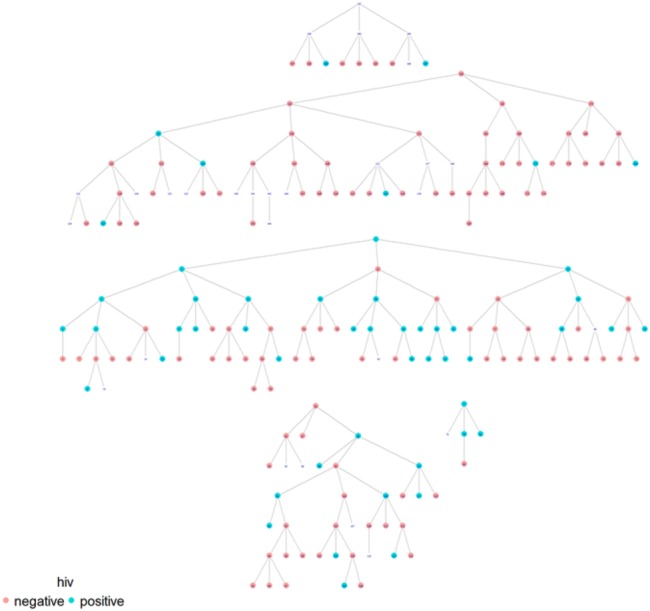
MSM network in Dar es Salaam by HIV status.

**Figure 4:**
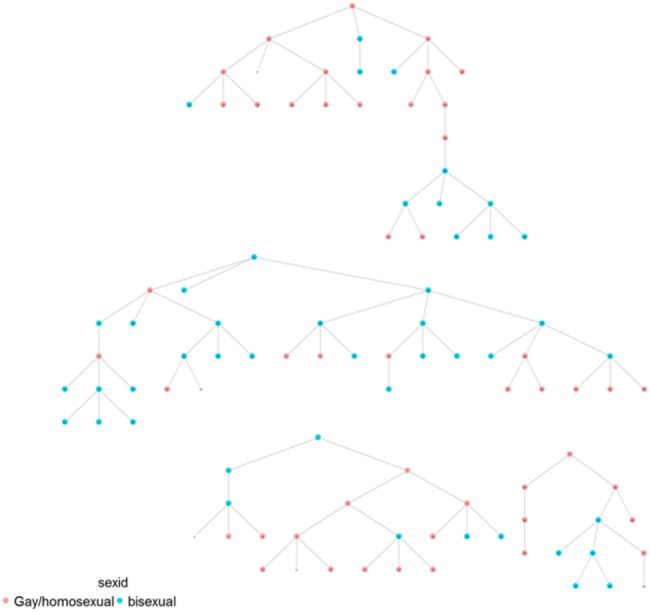
MSM network in Tanga by sexual identification.

As illustrated in table 5 homophile was significant for age (1.29; 1.07), ‘gay openness’ (1.15) and HIV status (1.66) in Dar es Salaam, while it was significant for sexual identification (1.25), solely in Tanga (figures 1–4). A value of 1 would indicate that there is no recruitment homophile, while a value of 2 would indicate that there are twice as many cases as we would expect if there was no homophile in the population.

**Table 5:** Recruitment homophile among MSM

Measure	Description	Dar es Salaam n=197		Tanga n=99	
		Homophile score	χ^2^ p value	Homophile score	χ^2^ p Value
Age grouping 1	18–21 vs 22–30 vs 31–59	1.29	**<0**.**001**	1.07	0.444
Age grouping 2	18–30 vs 31–59	1.07	**<0**.**001**	0.96	0.183
Education	Completed secondary vs below	1.03	0.642	0.98	0.838
Sexual identification	Gay/homosexual vs bisexual	1.09	0.108	1.25	**0**.**019**
Ever been paid in exchange for sex	Yes vs no	1.03	0.599	0.92	0.402
Stigma estimation in general community	Yes vs no	1.02	0.172	1.09	0.094
Stigma estimation among doctors, nurses, etc	Yes vs no	1.05	0.078	0.93	0.431
Gay openness	Openness index ≥0.5 vs <0.5	1.15	**0**.**042**	1.17	0.093
HIV status among those tested (laboratory test)	HIV positive vs HIV negative	1.66	**0**.**018**	1.03	0.369
STI status excluding HIV among those tested (laboratory test)	STI positive vs STI negative	1.01	0.679	NE	NE

Bold text indicates significance at p < 0.05.

STI was not estimable for Tanga due to high degree of cases with missing data (66%).

MSM, men who have sex with men; NE, not estimable; STI, sexually transmitted infection.

## Discussion

This study utilised RDS to recruit MSM in a small and a large city in Tanzania. The data provide unique perspectives on the social organisation of MSM and their networks, which could be used to inform interventions aiming to use RDS as a recruitment and/or dissemination strategy for HIV/STI prevention and treatment. As hypothesised, there was a significant difference between the size of the personal networks in the larger and the smaller city with larger and more specialised networks in Dar es Salaam compared with Tanga. The actual RDS networks followed a similar pattern. The analysis also revealed that homophile occurred in some of the comparisons made with more specialised recruitment patterns in the larger city than in the smaller city. The results indicate that RDS may be used as an effective strategy to leverage new or existing interventions that try to reach MSM in settings where homosexuality is criminalised and socially stigmatised.

Contrary to the hypothesis, almost the same number of seeds was actually required to reach minimum sample in Dar es Salaam (five seeds for n=200) than in Tanga (four seeds for n=100). Recruitment in this study was relatively slow in both cities, which could be explained by the nature of RDS studies. WHO/Joint United Nations Programme on HIV/AIDS, for example, recommend a 3–4-month time period for HIV surveillance surveys with 300 or more participants using RDS.^20^ In Tanga, the study brought in 100 participants in about 2 months (21 February to 16 April 2013) or 50 per month. In Dar es Salaam recruitment continued for 9 months (21 October to 27 July 2012). One possible explanation for the slow recruitment rate is that the parent study ran out of collection tubes for biological sampling in Dar es Salaam, which interrupted the data collection process for several months. However, there are also other factors that could have affected recruitment, such as incentive levels, community members' perceptions of the study, study site hours, and transportation barriers. Tanzania's anti-gay laws may be keeping these networks underground to avoid discrimination, victimisation, vigilante activity, and police harassment and arrest. A previous report conducted by Human Rights Watch found that MSM were frequently harassed by the police.^21^ Such incidents may affect participation rates in studies that rely on peer-driven recruitment, which emphasises the need for ample time planning when designing and implementing RDS studies in contexts with limited legal and social protection for sexual minorities.

The median reported personal network size, based on how many other MSM participants knew and had seen in the last month, was 6.0 in both Dar es Salaam and Tanga. In a study from Kampala on 224 MSM, participants reported a median of 30 MSM persons who they knew and had met during the past 6 months, while they estimated a median of 5 other MSM as close friends.^11^ Stahlman and colleagues found the median network size of 530 MSM in Lesotho and 322 in Swaziland to be 10 and 12, respectively.^22^ In the present study a large majority of the participants reported that they saw their recruiter at least once a month, and had known him for 1 year or more. Nearly all participants reported that they would have recruited the same person who had recruited them. Whether this is evidence of a high degree of cohesiveness in the networks cannot be ascertained based on available data. It is, however, an indication of reciprocity between the recruiter and recruited, which is an important prerequisite for trust between network members and critical for the outcome of an intervention that uses networks to reach participants.

*Actual* networks obtained with RDS showed mean sizes of 39.4 in Dar es Salaam and 25.3 in Tanga, consistent proportionally with the *reported* personal network sizes (12.0 and 7.6, respectively). The figures for the *actual* RDS networks include respondents further down the chain that would not necessarily be in the personal networks of others several waves up the chain and are hence much larger. The implication of such large networks from seeds is that peer educators may be able to reach large numbers of MSM using the total network structures. Given that networks were truncated when the sample n was reached, these figures describe networks that were likely smaller than the size would be if recruiting had continued.

The referral patterns indicate that significant homophile occurred in 5 of the 21 comparisons made, that is, sexual identification in Tanga, and for age, gay openness, and HIV status in Dar es Salaam. However, HIV homophile probably reflects disease transmission networks rather than aggregation by disease status knowledge, given that >90% of the confirmed HIV cases in the Dar es Salaam sample were *new* undiagnosed infections.^2^ While there is a risk that the homophile patterns identified were attributed to the risk associated with RDS to oversample individuals that are similar to the recruiter, the homophile patterns could also reflect the existence of relatively developed gay networks. As networks expand, MSM form further ties with others who have similar characteristics, which also is corroborated by other studies.^9^ ^23^ In smaller networks, on the other hand, options might be more limited, which is a possible interpretation of what our data demonstrate. An implication of these findings is that reaching MSM may require more specialised characteristics of seeds for recruitment, particularly in larger cities.

There are several limitations to this study. Some of the measurements used such as stigma estimation, sexual identification and sex work are subjected to cultural and societal contexts and definitions of these may vary across countries. This limits the generalisability of the results outside Tanzania. However, due to similar cultural and societal contexts within East Africa, it is possible that the results can be generalised to this region but requires empirical testing. Furthermore, given the stigma associated with homosexuality, it is likely that the initial sampling of seed individuals who were ‘out’ and part of a MSM subculture could have influenced subsequent recruitment chains, which might limit the study's representativeness.^16^ The convergence plots revealed that estimates of sexual orientation and gay openness in Dar es Salaam had not yet stabilised at the time when data collection ended, which may mean that the study under-represents bisexual MSM and over-represents openly gay men. If the recruitment process had continued for a longer period, those estimates could have continued to change, which would increase chances of reaching convergence for more measures. This raises important issues of seed selection as well as the number of waves of data collection when planning an RDS-based study or intervention. One strategy to address potential homophile bias could have been to use multiple selection of the initial seeds.^24^ Further in-depth qualitative and multinodal network research should be conducted to fully explore the composition of the MSM networks in these two cities.

These data have important implications for research and interventions with MSM in sub-Saharan African cities. It is apparent that even under conditions of heavy stigmatisation, there are MSM networks in both larger and smaller cities, with some selective affiliation into subgroups (homophile) developing in larger networks. Second, the existence of pre-existing contacts between recruiter and referral makes it possible to use peer-driven interventions to reach MSM in these networks, as has been done previously in a wide range of settings (although at perhaps a slower recruitment rate than in Western cities).^25–27^ Third, the personal network sizes imply that organisation (and presumably social support of other MSM) is present in the MSM community and may also be used to facilitate STI/HIV and community interventions in MSM in large and smaller sub-Saharan African cities. Our data also showed similarities with other RDS data from MSM in sub-Saharan Africa. Baral *et al*^28^ demonstrated in a study from Nigeria that RDS could be used as a method of engaging MSM in antiretroviral therapy services. Models of engagement of MSM such as the SPEND model ((P) Pharmacies as treatment sources; (E) Educate health professionals; (N) Navigation for patients who must access the health system; and (D) Discrimination reduction), which integrates the RDS approach to increase MSM awareness of available services that are trusted, access such networks.^29^ While these communities are to a large extent ‘underground’ for protection against stigmatisation and victimisation, there is a potential to connect large MSM networks to trusted healthcare workers through RDS or other network-based approaches.
